# Validation of a screening questionnaire for hip and knee osteoarthritis in old people

**DOI:** 10.1186/1471-2474-8-84

**Published:** 2007-08-23

**Authors:** José M Quintana, Inmaculada Arostegui, Antonio Escobar, Iratxe Lafuente, Juan C Arenaza, Isidoro Garcia, Urko Aguirre

**Affiliations:** 1 Unidad de Investigación, Hospital de Galdakao, Galdakao, Vizcaya, Spain; 2 Departamento de Matemática Aplicada, Estadística e Investigación Operativa, Universidad del País Vasco, Lejona, Spain; 3 Unidad de Investigación, Hospital de Basurto, Bilbao, Vizcaya, Spain; 4 Servicio de Traumatología, Hospital de Basurto, Bilbao, Vizcaya, Spain; 5 Servicio de Traumatología, Hospital de Galdakao, Galdakao, Vizcaya, Spain

## Abstract

**Background:**

To develop a sensitive and specific screening tool for knee and hip osteoarthritis in the general population of elderly people.

**Methods:**

The Knee and Hip OsteoArthritis Screening Questionnaire (KHOA-SQ) was developed based on previous studies and observed data and sent to 11,002 people aged 60 to 90 years, stratified by age and gender, who were selected by random sampling. Algorithms of the KHOA-SQ were created. Respondents positive for knee or hip OA on the KHOA-SQ were invited to be evaluated by an orthopedic surgeon. A sample of 300 individuals negative for knee or hip OA on the KHOA-SQ were also invited for evaluation. Sensitivity and specificity were determined for the KHOA-SQ, as well as for KHOA-SQ questions. Classification and Regression Tree analysis was used to find alternative screening algorithms from the questionnaire.

**Results:**

Of 11,002 individuals contacted, 7,577 completed the KHOA-SQ. Of 1,115 positive for knee OA, on the KHOA-SQ, 710 (63.6%) were diagnosed with it. For hip OA, 339 of the 772 who screened positive (43.9%) were diagnosed it. Sensitivity for the hip algorithm was 87.4% and specificity 59.8%; for the knee, sensitivity was 94.5% and specificity 43.8%. Two alternative algorithms provided lower specificity.

**Conclusion:**

The KHOA-SQ offers high sensitivity and moderate specificity. Although this tool correctly identifies individuals with knee or hip OA, the high false positive rate could pose problems. Based on our questions, no better algorithm was found.

## Background

Osteoarthritis (OA) of the hip and knee are common conditions that, in many cases, necessitate frequent follow-up, medical therapy, and potentially costly treatments, such as joint replacement surgery [1]. Nevertheless, relatively little is known about the prevalence of these pathologies in the general population [2]. Estimating prevalence requires tools that allow investigators to screen a large sample drawn from the general population to identify those individuals most likely to have the disease [3]. This can be a blood test, imaging modality, or other simple assay. None of these are yet available for hip or knee OA, although the search is underway for such tools [4,5]. Currently, investigators interested in determining the prevalence of hip or knee OA must rely on medical histories or descriptions of symptoms to identify individuals most likely to have the disease. Given that no single symptom can identify patients with hip or knee OA [6], the most relevant ones are often combined to construct an algorithm that ideally have high specificity and reasonable sensitivity [7]. Individuals identified by such screening tools as likely candidates must then undergo further evaluation so a definitive diagnosis can be made. Only the clinical evaluation of a patient with symptoms of knee or hip OA by an experienced physician and an x-ray of the joint can provide a definitive diagnosis.

We developed the Knee and Hip OsteoArthritis Screening Questionnaire (KHOA-SQ) as a possible tool for gauging the prevalence of hip and knee OA in a general population study.

## Methods

The study was conducted between April 2002 and December 2003 in the province of Vizcaya (Basque Country) in the north of Spain. Vizcaya has a population of 1,125,000 inhabitants, 23.6% of whom were aged 60 years or older. The province of Vizcaya is predominantly urban.

### Recruitment

To recruit participants from the general population, we used the register of the Basque Department of Health. This register includes all people covered by the National Health System which, in the Basque Country, covers almost 100% of the population. From that register, we performed a stratified random sampling by gender and age (3 categories: 60 to 69, 70 to 79, and 80 to 90 years) of all adults over sixty in the province.

Based upon a prevalence of 10% for knee OA and of 5% for hip OA observed in previous studies for patients aged 60 years or older [8-13], and using α = 0.05, 1-β = 0.8, and an error in the prevalence of OA of less than ± 1.5%, we estimated that we would need to recruit more than 7,000 individuals. Estimating a 10% exclusion rate and a participation rate of at least 70%, we planned to include at least 11,000 individuals in our study.

Selection criteria included: age ≥60 years, resident of Vizcaya, ability to complete the questionnaire and give consent to participate in the study, and ability to attend an outpatient clinic. We excluded individuals who were younger than 60 years, who lived outside the province, who did not have valid postal addresses or telephone numbers, who had severe psychiatric, sensorial or physical illness, or language problems that made it difficult to complete the questionnaire, or who were unable to attend the outpatient clinic.

### KHOA-SQ development

The KHOA-SQ was created to be a short, quickly completed questionnaire that included all relevant variables that alone or in combination would indicate, with a high sensitivity and specificity, possible OA of the knee or hip. To create the KHOA-SQ, we reviewed the available literature to determine if similar tools existed [3,9-12] and selected from prior studies the variables most likely to identify patients with knee or hip OA.

**Figure 1:**
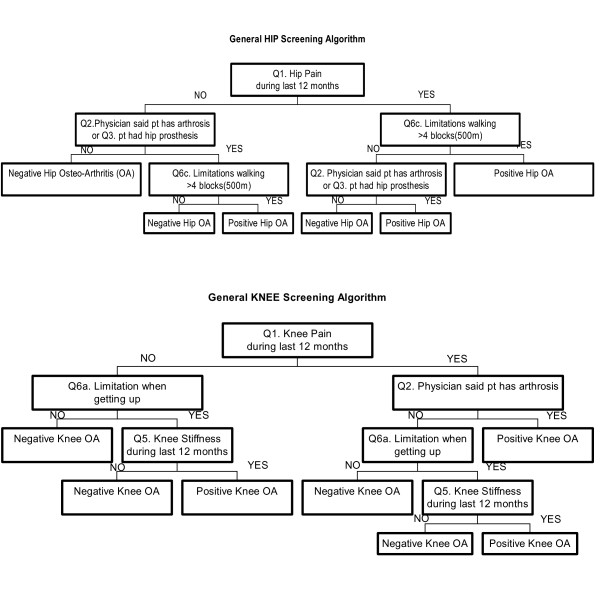
KHOA-SQ screening algorithms by articulation. Complete question is indicated in each square by the number of the question (Q#) of Tables 2 (knee) and 3 (hip) respectively.

The resulting questionnaire had 28 questions in three sections: 11 pertaining to knee osteoarthritis, 11 to hip osteoarthritis, and 6 common or general questions about both joints related to previous fractures, future interventions, and sociodemographic data. We conducted a pilot study with 400 patients undergoing urography or barium enema examinations to test which combination of questions offered the best balance of sensitivity and specificity for hip OA. The algorithms selected are presented in Figure 1. For the hip screening algorithm (algorithm #1, hip), we included 4 of the 11 original questions : "During the last 12 months, have you often had pain in one or both your hips for one month or more?"; "Has a doctor ever told you that you have osteoarthritis in one or both of your hips?"; "Do you have a prosthesis in one or both of your hips?"; and, "During the last 12 months, have you had frequent limitations or difficulties walking more than 4 blocks (500 m) because of pain or stiffness in one or both of your hips?"

For the knee screening algorithm (algorithm #1, knee), we also included 4 of the original 11 questions: "During the last 12 months, have you often had pain in one or both your knees for one month or more?"; "Has a doctor ever told you that you have osteoarthritis in one or both of your knees?"; "During the last 12 months, have you had any limitations rising from a chair or toilet because of pain or stiffness in one or both of your knees?"; and "During the last 12 months, have you had stiffness in one or both your knees for one month or more?" We based our election on previous studies [13].

In both cases, we created algorithms based on the previous questions to achieve the best possible sensitivity, while preserving reasonable specificity and positive predictive value (PPV). PPV positive was defined as OA real cases among those screening positive. Negative predictive value (NPV) is defined as no OA real cases among those screening negative. Sensitivity was defined as percentage of OA cases who screened positive, specificity as percentage of non-OA subjects who screened negative and accuracy as the percentage of screening results, both positive and negative, that were correct.

### Data collection

Letters were sent to 11,002 randomly selected residents of Vizcaya province to invite them to participate in the study. The letter presented the goals of the study, invited the recipient to participate, and asked for their informed consent. It also included the screening questionnaire for knee or hip OA along with a stamped return envelope. A reminder letter was sent to those who had not replied after 15 days. After 30 days, a copy of the questionnaire was sent to those who had not responded. Finally, we contacted by telephone those who had not responded within 45 days of the initial mailing.

We applied the algorithm to those who answered the KHOA-SQ. Individuals identified by the questionnaire as likely to have knee or hip OA were contacted by mail (up to three times, if necessary) or telephone to invite them to be evaluated for osteoarthritis at one of three hospitals chosen to provide ready access to the bulk of the population, and thus facilitate participation. Three orthopedic surgeons who collaborated in the study evaluated participants at any of the hospitals' outpatient clinics. These surgeons were trained by the research team and provided with standardized questionnaires requesting sociodemographic data, comorbidities, and symptoms (pain, stiffness, and functional limitations) and signs related to hip or knee osteoarthritis, separately. Information from a full clinical examination of the hip, knee, and lower back was also recorded. If the clinical examination was suggestive of hip or knee disease and the patient had not undergone an x-ray within the preceding 6 months, study participants were invited to have an x-ray of the affected joint(s). All x-rays were evaluated by the Kellgren and Lawrence [14] scale for hip OA and the Ahlbäck [15] scale for knee OA. Each orthopedic surgeon provided a final diagnosis about the presence or absence of OA. We classified individuals as having knee or hip OA if they had symptoms and radiographic evidence of OA in either of the hip or knee articulations. Patients also completed the Western Ontario and McMaster Universities Osteoarthritis Index (WOMAC) questionnaire[16,17].

We also included in the evaluation by the orthopaedic surgeons of some individuals who were negative for knee or hip OA on the KHOA-SQ. We randomly selected 300 individuals whose screening questionnaires were negative for both joints. Of these, 158 accepted and were reviewed by an orthopedic surgeon. Since the orthopedic surgeon evaluated all 4 joints for each participant, we included in the estimations those who screened negative for one part of the questionnaire (hip or knee joint) but were positive for the other. In all cases the orthopedic surgeons were blinded to the study goals and the results of the screening algorithm.

The study was approved by the hospitals Research Committees. All data were kept confidential.

### Statistical analysis

The unit of analysis was the person that was classified as positive OA for hip whenever any of his hips were positive and negative otherwise; or positive OA for knee whenever any of the knees were positive and negative otherwise. Frequencies and percentages were calculated as descriptive statistics of the sample. Hip and knee OA were treated separately.

In order to evaluate the possible presence of selection bias, we compared the answers on symptoms (see tables) to our screening questionnaire, by joint, between people who finally went, and those who did not, to the evaluation by the orthopaedic surgeon.

#### Evaluation of the KHOA-SQ

Selected OA screening algorithms for hip and knee were evaluated using sensitivity, specificity, and odds ratio by age and gender. For all of these estimates, 95% confidence intervals were calculated. Logistic regression models in which the dependent variable was the final diagnosis of kip or knee OA were used to estimate the c statistics of each question or algorithm, which were the independent variable in each model. In these analyses, the c statistic is a mathematical function of the sensitivity and specificity of our tool in classifying patients by means of a logistic regression model as either having OA or not. The c statistic is calculated as the fraction of patients with the outcome among pairs of patients where one has the outcome and one not, the patient with the highest prediction being classified as the one with the outcome (or c statistic estimate = .5 (1+Somer's D test). The null value for the C statistic is 0.5, with a maximum value of 1.0 (higher values indicating better).

Additionally, and as a way to provide with more information on the validity of our tool, we evaluated the KHOA-SQ results in relation to the WOMAC scores, by reported problems of hip and knee separately, only in those people who went to the revision by the orthopaedic surgeon and who completed the WOMAC. With those previous conditions, we were able to analyze 632 people who presented hip problems and 953 with knee problems. Means and standard deviations are provided. A Student *t *test was performed among those which gave positive for osteoarthritis, according to our KHOA-SQ, and those who did not.

Alternative screening algorithms to the KHOA-SQ were selected based on their sensitivity, specificity, positive predictive value, accuracy and odds ratio (OR: being OR the odds of having an OA). First, the association between individual questionnaire items and hip or knee OA was determined. Second, two alternative screening algorithms for hip and knee OA were created based on the ability of individual questions to predict OA. The simplest algorithm was based on two items, pain and previous OA status reported by any physician (algorithm #2). Another was based on three items related to OA symptoms – pain, stiffness, and the ability to walk more than 4 blocks (algorithm #3). Both were evaluated for hip and knee OA in the same sample.

Finally, we used Classification and Regression Tree (CART) analysis [18] to find the most informative way to classify subjects by their response to questionnaire items into successively more homogeneous groups with regard to symptomatic knee and hip OA. All the questions related to main symptoms (pain, stiffness, functional limitation, and insecurity [only for the knee]) and previous OA status reported by any physician were selected from the original questionnaire to find the optimal classification tree. Two different approaches of decision cost matrix were considered in the CART analysis. One penalized equally a false negative and a false positive, and the other penalized a false negative twice as much as a false positive. An optimal tree was created for hip OA with only pain and previous OA status reported by any physician, whereas for knee OA the items selected were limitation when going down steps or rising from a chair, previous OA status reported by any physician, and stiffness. Greater consistency between sensitivity and specificity was found when false negatives and false positives were penalized equally.

All effects were considered significant at p < 0.05 unless otherwise noted. Main statistical analyses were performed using SAS for Windows statistical software, version 8.2. (SAS Institute, Inc., Carey, NC). CART analysis was performed using CART with S-Plus software [19].

## Results

**Figure 2:**
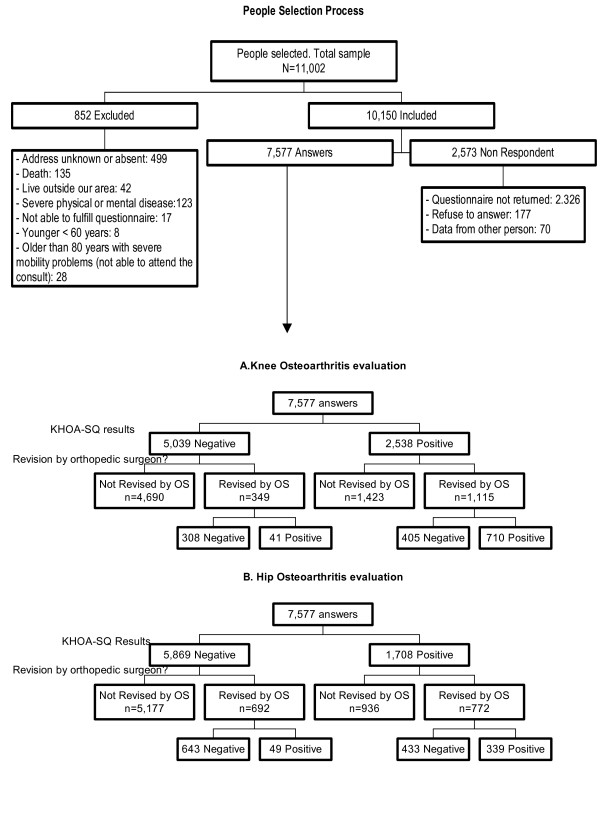
Patient selection process: number of patients included and excluded from the original sample.

Of the 11,002 randomly selected residents, 852 were excluded. Of the 10,150 finally included in the study, 2,326 did not return the KHOA-SQ after 3 mailed requests and a telephone contact, 177 explicitly refused to participate, and 70 provided information about a relative or other individual (Figure 2). The final participation rate was 74.65%.

The mean age of respondents was 71 years; 56.3% were women (Table 1). A total of 3,168 (41.8%) had KHOA-SQ results indicative for hip or knee OA, or both; knee OA was more common than hip OA. Of the 7,577 individuals who answered the KHOA-SQ, 1,115 of 2,538 with results indicative of knee OA were reviewed by an orthopedic surgeon. Of these, 710 (63.6%) were considered to have knee OA. With regard to hip OA, 772 of 1,708 respondents whose KHOA-SQ results were indicative of hip OA were reviewed by an orthopedic surgeon. Of these, 339 (43.9%) were considered to have hip OA (Figure 2). On the comparison to the answers on symptoms (those of tables 2 and 3) to our screening questionnaire, by joint, between people who finally went, and those who did not, to the evaluation by the orthopaedic surgeon we found that there were no statistically significant differences between both groups on presence of symptoms.

**Table 1:** Characteristics of individuals who answered the KHOA-SQ (n = 7577)^a^

	Total	Hip	Knee
Age: x¯ MathType@MTEF@5@5@+=feaafiart1ev1aaatCvAUfKttLearuWrP9MDH5MBPbIqV92AaeXatLxBI9gBaebbnrfifHhDYfgasaacH8akY=wiFfYdH8Gipec8Eeeu0xXdbba9frFj0=OqFfea0dXdd9vqai=hGuQ8kuc9pgc9s8qqaq=dirpe0xb9q8qiLsFr0=vr0=vr0dc8meaabaqaciaacaGaaeqabaqabeGadaaakeaacuWG4baEgaqeaaaa@2E3D@ (DE)	70.96 (6.96)		
<70	3730 (49.23)		
70–79	2886 (38.09)		
≥ 80	961 (12.68)		
Gender:			
Male (%)	3313 (43.72)		
Female (%)	4264 (56.28)		
KHOA-SQ screening positive: n (%)	3168 (41.81)	1708 (22.54)	2538 (33.50)

^a ^Percentages were calculated based on non missing answers.

KHOA-SQ = Knee and Hip OsteoArthritis Screening Questionnaire

**Table 2:** Rate of response and association of responses to individual questions about knee OA with symptomatic knee osteoarthritis (n = 1464)

Question	**n**	Frequency of positive findings, %	Sensitivity,% (95% CI)	Specificity,% (95% CI)	Positive Predictive Value,% (95% CI)	Negative Predictive Value,% (95% CI)	Odds ratio (95% CI)	C statistics
**1. **– During the last 12 months, have you often had pain in one or both your knees for one month or more?	1413	80.96	94.77 (93.17, 96.37)	34.48 (30.87, 38.09)	61.80 (58.98, 64.62)	85.50 (81.29, 89.71)	9.54 (9.66, 13.67)	0.646
**2. **– Has a doctor ever told you that you have osteoarthritis in one or both of your knees?	1419	62.86	80.70 (77.87,83.53)	56.91 (53.17, 60.65)	67.49 (64.42, 70.56)	72.68 (68.88, 76.48)	5.52 (4.35, 7.00)	0.688
**3. **– Do you have a prosthesis in one or both of your knees?	1414	3.75	6.76 (4.95, 8.57)	99.55 (99.04,100.1)	94.34 (88.12, 100.6)	49.30 (46.64, 51.96)	16.21 (5.03, 52.21)	0.532
**4. **– Have you had any surgical intervention in one or both of your knees?	1415	11.38	16.40 (13.73, 19.07)	94.09 (92.31, 95.87)	75.16 (68.49, 81.83)	50.80 (48.03, 53.57)	3.12 (2.15, 4.54)	0.552
**5. **– During the last 12 months, have you had stiffness in one or both your knees for one month or more?	1417	48.98	64.37 (60.92, 67.82)	67.90 (64.38, 71.42)	68.73 (65.28, 72.18)	63.49 (59.98, 67.00)	3.82 (3.07, 4.77)	0.661
**6. **– During the last 12 months, have you had any of the following limitations because of pain or stiffness in one or both of your knees?								
**6 a. **– Rising from a chair or toilet	1360	61.91	78.58 (75.58, 81.58)	56.79 (52.96, 60.62)	67.10 (63.93, 70.27)	70.27 (66.33, 74.21)	4.82 (30.80 6.11)	0.677
**6 b. **– Going up steps	1380	69.71	85.44 (82.88, 88.00)	47.85 (44.02, 51.68)	64.66 (61.64, 67.68)	74.64 (70.47, 78.81)	5.38 (4.16, 6.96)	0.666
**6 c. **– Going down steps	1374	70.74	88.05 (85.69, 90.41)	48.76 (44.91, 52.61)	65.95 (62.97, 68.93)	78.36 (74.33, 82.39)	7.01 (5.34, 9.20)	0.684
**6d. **– Walking 4 blocks (or 500 m)	1348	55.34	70.72 (67.37, 74.07)	61.62 (57.86, 65.38)	67.02 (63.65, 70.39)	65.61 (61.82, 69.40)	3.88 (3.09, 4.87)	0.662
**7. **– During the last 12 months, have you often experienced any sensation of insecurity or that one or both of your knees failed?	1394	62.55	77.05 (74.00, 80.10)	53.47 (49.67, 57.27)	64.68 (61.51, 67.85)	67.82 (63.81, 71.83)	3.86 (3.07, 4.86)	0.653

95% CI = 95% confidence interval

c statistics: estimated by logistic regression model

**Table 3:** Rate of response and association of responses to individual questions about hip OA with symptomatic hip osteoarthritis (n = 1464)

Question	**n**	Frequency of positive findings,%	Sensitivity,% (95% CI)	Specificity,% (95% CI)	Positive Predictive Value,% (95% CI)	Negative Predictive Value,% (95% CI)	Odds ratio (95% CI)	C statistics
**1. **– During the last 12 months, have you often had pain in one or both your hips for one month or more?	1316	56.69	87.09 (83.65, 90.53)	54.94 (51.78, 58.10)	42.49 (38.94, 46.04)	91.75 (89.49, 94.01)	8.22 (5.90, 11.46)	0.712
**2. **– Has a doctor ever told you that you have osteoarthritis in one or both of your hips?	1357	40.01	66.67 (61.92, 71.42)	70.28 (67.42, 73.14)	46.41 (42.22, 50.60)	84.52 (82.04, 87.00)	4.73 (3.67, 6.10)	0.685
**3. **– Do you have a prosthesis in one or both of your hips?	1362	4.92	16.04 (12.32, 19.76)	99.29 (98.77, 99.81)	89.55 (82.23, 96.87)	75.75 (73.42, 78.08)	26.78 (12.12, 59.19)	0.577
**4. **– Have you ever had any surgical intervention in one or both of your hips?	1366	3.07	8.22 (5.45, 10.99)	98.89 (98.24, 99.54)	73.81 (60.50, 87.12)	73.87 (71.50, 76.24)	7.97 (3.96, 16.02)	0.536
**5. **– During the last 12 months, have you had stiffness in one or both of your hips for one month or more?	1352	28.48	47.03 (41.94, 52.12)	78.51 (75.94, 81.08)	45.19 (40.22, 50.16)	79.73 (77.20, 82.26)	3.24 (2.51, 4.18)	0.628
**6. **– During the last 12 months, have you had any of the following limitations because of pain or stiffness in one or both of your hips?								
**6.a. **– Rising from a chair or toilet.	1331	45.98	69.92 (65.24, 74.60)	63.20 (60.15, 66.25)	42.16 (38.25, 46.07)	84.56 (81.92, 87.20)	3.99 (3.08, 5.17)	0.666
**6 b. **– Putting on your stockings or shoes.	1336	47.68	72.85 (68.33, 77.37)	62.03 (58.97, 65.09)	42.54 (38.70, 46.38)	85.55 (82.94, 88.16)	4.38 (3.37, 5.70)	0.674
**6 c. **– Walking 4 blocks (or 500 m)	1326	42.76	69.11 (64.40, 73.82)	67.40 (64.43, 70.37)	44.97 (40.88, 49.06)	84.98 (82.44, 87.52)	4.62 (3.57, 5.99)	0.683

95% CI = 95% confidence interval

c statistics: estimated by logistic regression model

Of the final sample of 1,464, including those positive and negative to the KHOA-SQ, from whom we have information about the definite diagnosis, 934 were women and 530 men; 840 were between the ages of 60 and 69, 525 were between 70 and 79, and 99 were aged 80 years or older.

We analyzed all relevant individual variables included in the KHOA-SQ to identify associations with a final diagnosis of OA. For knee OA, the questions with the highest specificity were " Has a doctor ever told you that you have osteoarthritis in one or both of your knees?" (99.5%), "Have you had any surgical intervention in one or both of your knees" (94.1%), and " During the last 12 months, have you had stiffness in one or both your knees for one month or more?" (67.9%). The questions with the highest sensitivity were "During the last 12 months, have you often had pain in one or both your knees for one month or more?" (94.8%), and " During the last 12 months, have you had any limitations going up (85.4%) or down (88%) stairs because of pain or stiffness in one or both of your knees?" (Table 2).

For hip OA, the questions with the highest specificity were " Do you have a prosthesis in one or both of your hips?" (99.3%), " Have you ever had any surgical intervention in one or both of your hips?" (98.9%), and "During the last 12 months, have you often had pain in one or both your hips for one month or more?" (78.5%) (Table 3). The questions with the highest sensitivity were "During the last 12 months, have you often had pain in one or both your hips for one month or more?" (87.1%), followed by " During the last 12 months, have you had any limitations on your stockings or shoes because of pain or stiffness in one or both of your hips?" (72.8%).

In relation to the study of the correlation of our KHOA-SQ with the WOMAC, the results were as follows. For people who, according to the KHOA-SQ, did not seem to have problems of OA in anyone of their hips the mean score in the 3 WOMAC domains (pain, stiffness, and functional limitation) measured by the WOMAC were, respectively, 25.1 (18), 23.9 (22.2) and 25 (19.8), whereas for those that, according to the KHOA-SQ, may suffer of OA the means were 38.5 (20.5), 37.7 (24.6) and 40 (20.1) respectively in the WOMAC domains. In the case of the knee, the means were 19.1 (15.8), 21. 4 (22.3), and 23.2 (21.7) for those who did not seem to have OA problems; whereas in the case of those who were suggestive to suffer OA problems the means were, respectively, 36.5 (19.7), 35 (25), and 38.2 (20.8). In all the cases, the differences between those which were positive for OA, according to our KHOA-SQ, and those who were not, were statistically significant with p < 0.001 in all the conducted comparisons.

The knee OA screening algorithm we developed had a sensitivity of 94.5%, a specificity of 43.2%, a positive predictive value (PPV) of 63.7%, a negative predictive value (NPV) of 88.3%, an accuracy of 69.5%, and a c statistic of 0.69. The hip OA screening algorithm had a sensitivity of 87.4%, a specificity of 59.8%, a PPV of 43.9%, an NPV of 92.9%, an accuracy of 67.1%, and a c statistic of 0.74. We examined the effect of age and gender on these metrics (Table 4). For the knee OA algorithm, sensitivity and specificity were similar in all age classes except for men aged 80 and older, in whom the specificity dropped considerably. Differences by gender were minor. For the hip OA algorithm, sensitivity and specificity were again similar by age, except among older men, in whom the sensitivity dropped moderately. The sensitivity was slightly better for women, while the specificity was better for men.

**Table 4:** Sensitivity, specificity and odds ratios (OR) of the screening questionnaire for knee or hip osteoarthritis by gender and age

		**Sensitivity,% (95% CI)**	**Specificity,% (95% CI)**	**OR (95% CI)**
**Hip**	Total	87.37% (85.03, 89.71)	59.76% (56.11, 63.41)	10.27 (7.43, 14.19)
**Men:**	< 70	86.11% (78.12, 94.10)	67.65% (61.71, 73.59)	12.96 (6.30, 26.66)
	70–79	83.02% (72.91, 93.13)	62.86% (54.86, 70.86)	8.27 (3.74, 18.32)
	≥ 80	75.00% (50.50, 99.50)	73.33% (50.95, 95.71)	8.25 (1.45, 46.86)
				
**Women:**	< 70	89.68% (84.37, 94.99)	54.46% (49.60, 59.32)	10.39 (5.67, 19.06)
	70–79	90.82% (85.10, 96.54)	57.26% (50.92, 63.60)	13.29 (6.37, 27.58)
	≥ 80	81.48% (66.83, 96.13)	64.44% (50.45, 78.43)	7.98 (2.54, 25.11)

**Knee**	Total	94.54% (93.21, 95.87)	43.20% (38.00, 48.40)	13.17 (9.29, 18.65)
**Men:**	< 70	95.83% (92.25, 99.41)	42.11% (35.09, 49.13)	16.73 (6.53, 42.85)
	70–79	95.24% (91.17, 99.31)	45.45% (35.05, 55.85)	16.67 (6.18, 44.91)
	≥ 80	93.33% (80.70, 100)*	16.67% (0, 37.76)*	2.80 (0.22, 35.29)
				
**Women:**	< 70	92.51% (89.35, 95.67)	45.63% (39.61, 51.65)	10.36 (6.18, 44.91)
	70–79	96.43% (93.83, 99.03)	40.44% (32.19, 48.69)	18.33 (8.01, 41.98)
	≥ 80	93.75% (86.90, 100)*	45.83% (25.90, 65.76)	12.69 (3.07, 52.40)

95% CI = 95% confidence interval. *Lower and upper limits have been set up to 0 and 100 when they were negative or greater than 100, respectively.

Since the construction of our negative rows for the calculations of the sensitivity and specificity came from different sources (those evaluated as negatives and that had reported negative both of the screening algorithms (hip/knee) and those evaluated as negatives and that had reported negative to one of the screening algorithms (hip or knee)) we studied the presence of some bias. In both joints, the analysis between those evaluated as negatives and that had reported negative both of the screening algorithms (hip/knee) versus those who were negative to the explored joint but not the other on relation to the final diagnosis of osteoarthritis showed not statistically significant differences in both joints for those groups.

**Figure 3:**
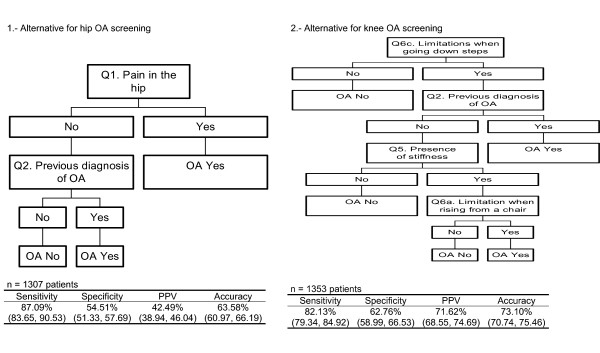
Alternative screening algorithms for each joint. In parenthesis 95% confidence intervals. Complete question is indicated in each square by the number of the question (Q#) of Tables 2 (knee) and 3 (hip) respectively

We examined whether alternative algorithms could achieve better sensitivity or specificity. None of those evaluated (algorithms #2 and 3), offered improved sensitivity or specificity. We also used the CART analysis to select additional algorithm trees for knee or hip OA. In the case of hip OA, the single question "During the last 12 months, have you often had pain in one or both your hips for one month or more?" had a reasonable good sensitivity (87.1%) though a relatively low specificity (54.9%). A simple algorithm that included only two questions about the presence of pain and a diagnosis of OA by a physician) provided similar sensitivity (87.1%) and specificity (54.5%) (Figure 3). The CART knee algorithm provided with a better specificity (62.8%) and worse sensitivity (82.1%) than our algorithm (Algorithm #1), and included 4 variables in a more complex algorithm.

## Discussion

In the absence of a simple screening test for knee or hip OA [20], determining the prevalence in a general population is difficult. Basically, the symptoms referred by the patient are the key issue to screening. We developed a screening questionnaire for identifying individuals with knee or hip OA with high sensitivity and moderate specificity.

Accurate data on the prevalence and severity of knee and hip OA in the general population are needed for a variety of reasons. In many cases, patients present with severe pain and functional limitation of the affected joint, which often translates into a diminished quality of life [21]. Accurate prevalence data can help health care systems allocate resources for the costly medical and surgical care, as well as rehabilitation programs, needed to manage and treat patients with knee and hip OA [13,22,23].

Our study is not the first to attempt to ascertain the symptoms most likely to identify individuals with knee or hip OA [3,9-12,24]. However, to the best of our knowledge it is the first to evaluate different algorithms of symptoms for both joints together. Several simple or complex algorithms have been developed to screen for knee OA [3,10,12,24] or hip OA [9,11] in the general population. In fact, we based the selection of variables for our KHOA-SQ on findings of these studies. In the case of hip OA, prior investigators had suggested "During the last 12 months, have you often had pain in one or both your hips for one month or more?" as the unique question to identify individuals with this condition [9]. We found that this single question has good sensitivity, though low specificity. Screening for knee OA appears to be more complex, as we and others [3] have shown. The question "During the last 12 months, have you often had pain in one or both your knees for one month or more?" was not nearly as sensitive as was the corresponding question about hip pain. A selection of the most predictive questions by CART analysis provided a stronger algorithm, though a more complex one that was far from perfect.

Studies on the prevalence of knee and hip OA have been conducted in all parts of the world, including the UK [9-12,24], the US [3,25], Canada [13], Europe (specifically Sweden, Denmark, Iceland, Netherlands, and Italy) [26-30], Australia [31], China [32,33], and Saudi Arabia [34]. In most of these studies, screening – when it was conducted – used a simple question such as "Do you have pain in a particular joint?" followed by a complete examination of those reporting such pain by an orthopedic surgeon, as was done in our study. In contrast, we employed more questions in a more complex algorithm to screen for individuals with knee or hip OA.

The main limitation of our study is missing data. Despite three mailings and a telephone contact, one-quarter of the study population did not complete or return the screening questionnaire. Even so, our response rate (74.65%) was similar to or better than response rates achieved in previous studies. Among the respondents identified by the KHOA-SQ as being positive for knee or hip OA, 55–56% did not attend an appointment for evaluation by one of the orthopedic surgeons involved in the study. We aimed to make these appointments as convenient as possible by asking the respondent to choose an opportune day and time, and the surgeons saw patients in clinics that were accessible by public transportation and that offered a wide geographic range. Even so, some of the respondents would have had to travel up to 50 kilometers to be evaluated. Those without a relative or friend to help with transportation may have found this a difficult task. It is possible that respondents who did not come for evaluation were more likely to have mobility problems due to OA. This fact may have also biased our estimations of sensitivity and specificity, though, as we have shown, no differences were found in the rate of symptoms among attendants and non attendants.

An additional problem is the composition of the group of negative result to our screening algorithm that was evaluated by the orthopaedics surgeons. It contained people who gave negative for both joints and people who gave negative to the symptoms of the evaluated joint but not to the other. This may have biased our results. Nevertheless, the rates of those diagnosed finally as osteoarthritis between both groups for each joint were not different statistically.

Another different and important issue is the presence of a spectrum bias in our study. This comes from the different rate from negative/positive test between the data found in the field work (test positive 33% for knee and 22. 5% for hip- Figure 2) and the configuration of the 2 × 2 table for the estimation of sensitivity and the specificity. The number of included for the positive/negative test rows for the estimation of sensitivity and the specificity did not respect those percentages (76.2/23.8 for the knee and 52.7/47.3% for the hip respectively). This do have implications on the estimation of the sensitivity and the specificity, being, possible that the real sensitivity is lower and higher the specificity.

The performance of the OA evaluations by three different three orthopedic surgeons may have been another limitation of the study. Differential assessments by each of the surgeons could have biased the results. However, all three were trained before the study, were asked to conduct each examination according to a prepared manual, and completed a standardized questionnaire to gather identical information for each patient. For making a definitive diagnosis of knee or hip OA, we followed criteria established by several scientific societies [35,36], based on the patient's self-reported symptoms and evaluation of an x-ray. In addition the surgeons were blinded as to the study goals.

Finally, our study population included only individuals aged 60 and older, thus our results have no generalizability to a younger population.

The screening algorithms we defined have, in general, acceptable sensitivity but poor specificity. Their low positive predictive value and the high rate of false positives have some consequences. With the current estimations of the sensitivities and specificities of our screening algorithm false positives may outweighing true positives. If this happens, it may be an important overload for any sanitary service or may require furtherscreening to avoid unnecessary exams. In any case, our real rate of positive tests in this field work was of 33 of and a 22.5% (knee and hip). From the health system perspective, use of such algorithms would necessitate the evaluation of several patients without knee or hip OA for each one definitively diagnosed with these conditions, something usual on screening tools. From the patient's point of view, a positive screening test could be stressful, but given that knee and hip OA are usually not potentially fatal conditions, the stress may not be as serious as it would be for a positive test for lung cancer, for instance.

## Conclusion

The development of a screening algorithm for knee or hip OA that provides adequate sensitivity and specificity is an important goal for the management of these conditions [3]. Our algorithm, which with the current results seem to be sensitive but not highly specific for knee or hip OA, can be used in screening studies. At the present time, screening the general population for knee and hip OA must still depend on assessment of individuals' self-reported symptoms followed by clinical evaluation. But given the importance of learning more about the real prevalence and impact of knee and hip OA, even imperfect tools such as the KHOA-SQ have a place in epidemiological studies. Nevertheless, an important issue for the future would be to test the KHOA-SQ in other settings with complete data for those positive and negative to the test.

## Competing interests

The author(s) declare that they have no competing interests.

## Authors' contributions

JMQ, IA and AE conceived of the study, coordinated and participated in the design of the study, and drafted the manuscript. JMQ, AE, IL, JCA and IG participated in the design, data collection and helped draft the manuscript. IA and UA performed the statistical analyses and drafted the manuscript. All authors read and approved the final manuscript.

## Pre-publication history

The pre-publication history for this paper can be accessed here:


